# Supplementary material for: Ventromedial frontoinsular connectivity is associated with long-term smoking behavior change in aging
Source: Imaging Neurosci (Camb). 2024 May 9;2:imag-2-00142. doi: 10.1162/imag_a_00142 (PMC12247603; doi:10.1162/imag_a_00142)
Supplement: Supplementary Material [file imag_a_00142-supp.pdf]

Ventromedial Frontoinsular Connectivity
is Associated with Long-term Smoking Behavior Change in Aging

Supplementary Materials

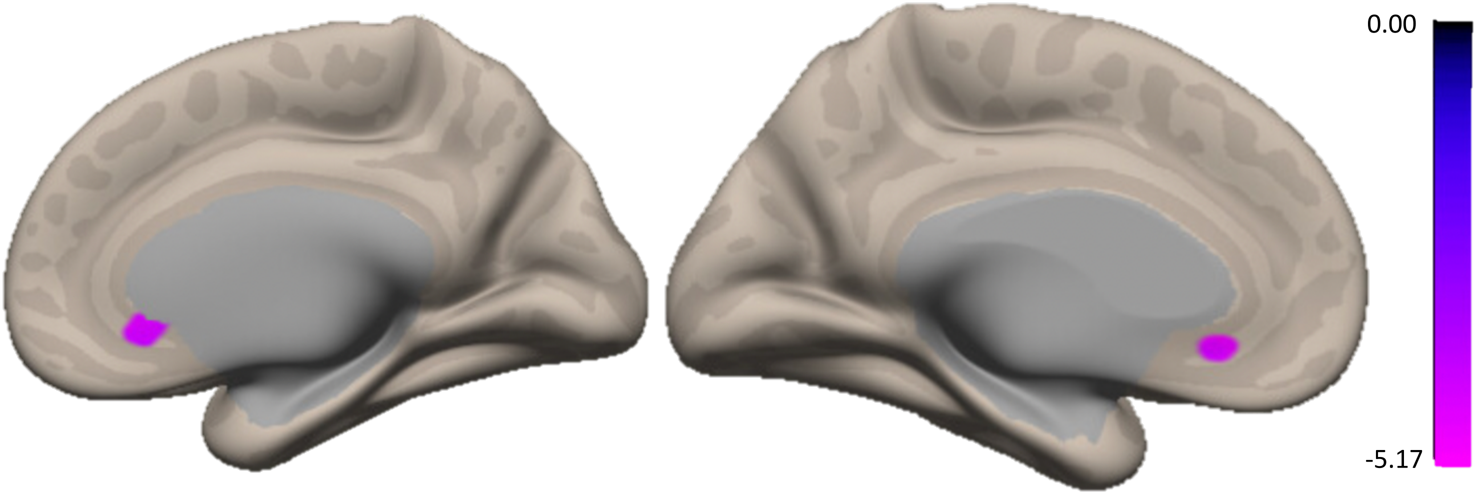

Supplementary figure 1. This figure displays the results from the seed-to-voxel analysis using a more stringent head motion threshold and subsequently reduced sample size. We repeated data preprocessing with the more conservative 0.5mm scan-to-scan framewise displacement threshold per one reviewers' suggestion. Maintaining the minimum of 5 minutes of scan time resulted in 5 participants needing to be removed from the PREVENT-AD sample (leaving a sample of N=18), and 17 from the UK BioBank (leaving a sample of N=100). Given the significant reduction in the sample size we performed a power analysis (in python) on our sample size to ensure sufficient power was gained to detect a true effect. Based on our FC MRI sample size (N=23) and an assumed type I error rate of 0.05, we calculated an estimated 81% power to detect an effect size of 0.11 from the seed-to-voxel F test. With the reduced sample size of N=18 and the same assumptions, we calculated an estimated 61% power. The subcallosal cortex cluster in this reduced sample (voxel height $p<0.001$ uncorrected; FDR cluster-level $p<0.05$ correction; k =30 voxels; Peak MNI voxel = +00, +30, -08 [x, y, z coordinates]) is maintained consistent with clusters found within the entire fMRI sample of N=23.

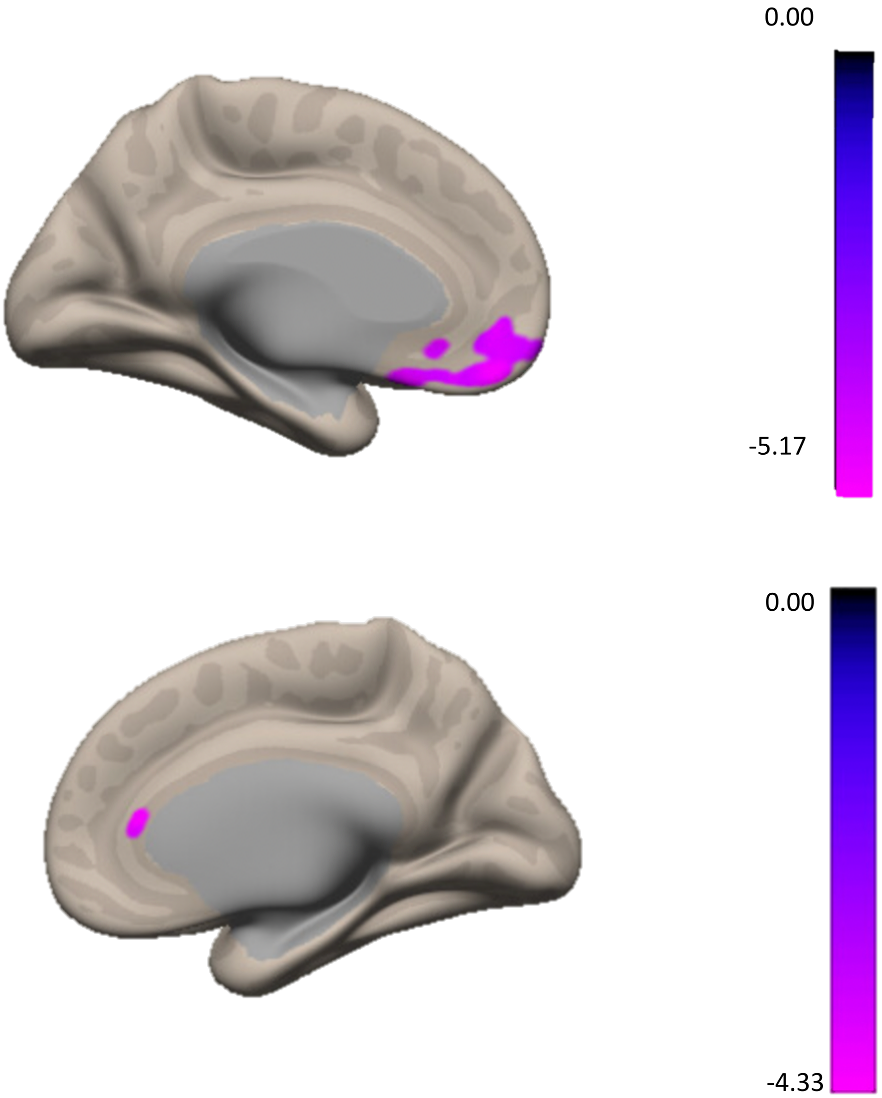

Supplementary Figure 2. **Functional connectivity results from the bilateral anterior insula seed after excluding participants who increased their smoking**. Seed-to-voxel resting-state functional connectivity results in the PREVENT-AD sample (a): Decreased smoking at follow-up compared to baseline was associated with diminished functional connectivity between bilateral anterior insula and a cluster within ventromedial prefrontal cortex (vmPFC) (a), with a peak voxel was located within subcallosal cortex ($F(2, 17) = 5.60$; voxel height $p<0.001$ uncorrected; cluster p-FWE <0.05; $k = 178$ voxels; Peak MNI voxel = +40, +30, -10 [x, y, z coordinates]). Seed-to-voxel resting-state functional connectivity results in the UK-Biobank sample (b): Decreased smoking in follow-up compared to baseline was associated with lower functional connectivity between bilateral anterior insula and a cluster within the ventromedial prefrontal cortex (vmPFC), with a peak voxel located within the anterior cingulate cortex ($F(2, 13) = 10.58$; voxel height $p<0.001$ uncorrected; cluster p-FWE <0.05; $k = 75$ voxels; Peak MNI

voxel = +06, +34, +12 [x, y, z coordinates]). Connectivity results are overlaid on the MNI template brain and corrected for multiple comparisons. Age, sex, baseline smoking amount, *APOE4* carrier status, and mean head motion were used as covariates of non-interest, as in the initial analysis.
